# Targeting Pro-Oxidant Iron with Deferoxamine as a Treatment for Ischemic Stroke: Safety and Optimal Dose Selection in a Randomized Clinical Trial

**DOI:** 10.3390/antiox10081270

**Published:** 2021-08-10

**Authors:** Mònica Millán, Núria DeGregorio-Rocasolano, Natàlia Pérez de la Ossa, Sílvia Reverté, Joan Costa, Pilar Giner, Yolanda Silva, Tomás Sobrino, Manuel Rodríguez-Yáñez, Florentino Nombela, Francisco Campos, Joaquín Serena, José Vivancos, Octavi Martí-Sistac, Jordi Cortés, Antoni Dávalos, Teresa Gasull

**Affiliations:** 1 Department of Neurosciences, Hospital Germans Trias i Pujol, 08916 Badalona, Barcelona, Spain; ndgregorio@igtp.cat (N.D.-R.); nperez.germanstrias@gencat.cat (N.P.d.l.O.); silvia.reverte@urv.cat (S.R.); adavalos.germanstrias@gencat.cat (A.D.); 2 Cellular and Molecular Neurobiology Research Group, Department of Neurosciences, Germans Trias i Pujol Research Institute (IGTP), 08916 Badalona, Barcelona, Spain; omarti@igtp.cat or; 3 Department of Clinical Pharmacology, Hospital Germans Trias i Pujol, 08916 Badalona, Barcelona, Spain; joan.costa.pages@gmail.com; 4 Department of Pharmacy, Hospital Germans Trias i Pujol, 08916 Badalona, Barcelona, Spain; pginerb@gmail.com; 5 Department of Neurology, Hospital Dr. Josep Trueta, 17007 Girona, Spain; ysilva.girona.ics@gencat.cat (Y.S.); jserena.girona.ics@gencat.cat (J.S.); 6 Clinical Neurosciences Research Laboratory, Health Research Institute of Santiago de Compostela, Hospital Clínico Universitario, Universidade de Santiago de Compostela, 15706 Santiago de Compostela, Spain; tomas.sobrino.moreiras@sergas.es (T.S.); Francisco.Campos.Perez@sergas.es (F.C.); 7 Department of Neurology, Hospital Clínico Universitario, 15706 Santiago de Compostela, Spain; manuel.rodriguez.yanez@sergas.es; 8 Department of Neurology, Hospital La Princesa, 28006 Madrid, Spain; fnombela.hlpr@salud.madrid.org (F.N.); joseaurelio.vivancos@salud.madrid.org (J.V.); 9 Department of Cellular Biology, Physiology and Immunology, Universitat Autònoma de Barcelona, 08193 Bellaterra, Spain; 10 Department of Statistics and Operations Research, Universitat Politècnica de Catalunya (UPC), 08028 Barcelona, Spain; jordicortes40@gmail.com

**Keywords:** iron, deferoxamine, antioxidant, ferroptosis, neuroprotection, outcome

## Abstract

A role of iron as a target to prevent stroke-induced neurodegeneration has been recently revisited due to new evidence showing that ferroptosis inhibitors are protective in experimental ischemic stroke and might be therapeutic in other neurodegenerative brain pathologies. Ferroptosis is a new form of programmed cell death attributed to an overwhelming lipidic peroxidation due to excessive free iron and reactive oxygen species (ROS). This study aims to evaluate the safety and tolerability and to explore the therapeutic efficacy of the iron chelator and antioxidant deferoxamine mesylate (DFO) in ischemic stroke patients. Administration of placebo or a single DFO bolus followed by a 72 h continuous infusion of three escalating doses was initiated during the tPA infusion, and the impact on blood transferrin iron was determined. Primary endpoint was safety and tolerability, and secondary endpoint was good clinical outcome (clinicalTrials.gov NCT00777140). DFO was found safe as adverse effects were not different between placebo and DFO arms. DFO (40–60 mg/Kg/day) reduced the iron saturation of blood transferrin. A trend to efficacy was observed in patients with moderate-severe ischemic stroke (NIHSS > 7) treated with DFO 40–60 mg/Kg/day. A good outcome was observed at day 90 in 31% of placebo vs. 50–58% of the 40–60 mg/Kg/day DFO-treated patients.

## 1. Introduction

A growing body of novel evidence points to a newly described type of programmed cell death having a pivotal role in neurodegeneration. This cell death is known as ferroptosis, which is iron- and lipid peroxidation-dependent, and associated with reduced cellular antioxidant activity. In support of this concept, ferroptosis has been recently reported to drive the acute neurodegeneration observed in ischemic and hemorrhagic stroke [1,2,3,4,5,6]; in traumatic brain injury [7]; or in long-term neurodegeneration observed in pathologies such as Alzheimer’s disease, Parkinson’s disease, or Huntington’s disease [8].

Preclinical investigations indicate that iron, either resident in brain cells, released from hemolyzed red blood cells that reach the brain parenchyma during hemorrhage, imported from blood to brain as free-labile iron or by iron-carrying molecules, or acting at the cerebral vasculature, is a potent pro-oxidant that contributes to the neurodegeneration and brain damage observed in acute ischemic stroke (AIS) and in intracerebral hemorrhage (ICH) [4,6,8,9,10,11,12]. Iron exacerbates excitotoxicity and induces neurodegeneration through the production of highly reactive and cytotoxic hydroxyl radicals which foster oxidative DNA damage and lipid peroxidation [12,13,14]. In the clinical arena, iron overload conditions at admission, measured as high ferritin levels in blood, have been consistently associated with poor functional outcome in patients with either intracranial hemorrhage [15,16] or ischemic stroke [17,18]. Moreover, increased systemic iron stores are associated with severe edema and with symptomatic hemorrhagic transformation in ischemic stroke patients treated with thrombolytic reperfusion therapy with intravenous recombinant tissue plasminogen activator (tPA) [18]. Importantly, the biological iron chelator and powerful antioxidant deferoxamine mesylate (DFO), that has long been used in clinic as a first line treatment to remove excess iron in iron-overload diseases such as thalassemia [19], has demonstrated its effectiveness as a neuroprotective agent in experimental stroke models of AIS and ICH [4,20,21,22,23] and prevents the excess of mitochondrial free radicals induced in transient ischemic stroke models [24].

Given the pivotal role of iron in reactive oxygen species production, ferroptosis, and ischemia/reperfusion damage, and since iron chelation with DFO is neuroprotective in experimental stroke models and well-tolerated in ICH patients [25], the TANDEM 1 study aimed to evaluate safety and tolerability, and to explore potential efficacy of intravenous DFO administered to ischemic stroke patients.

## 2. Materials and Methods

**Figure 1:**
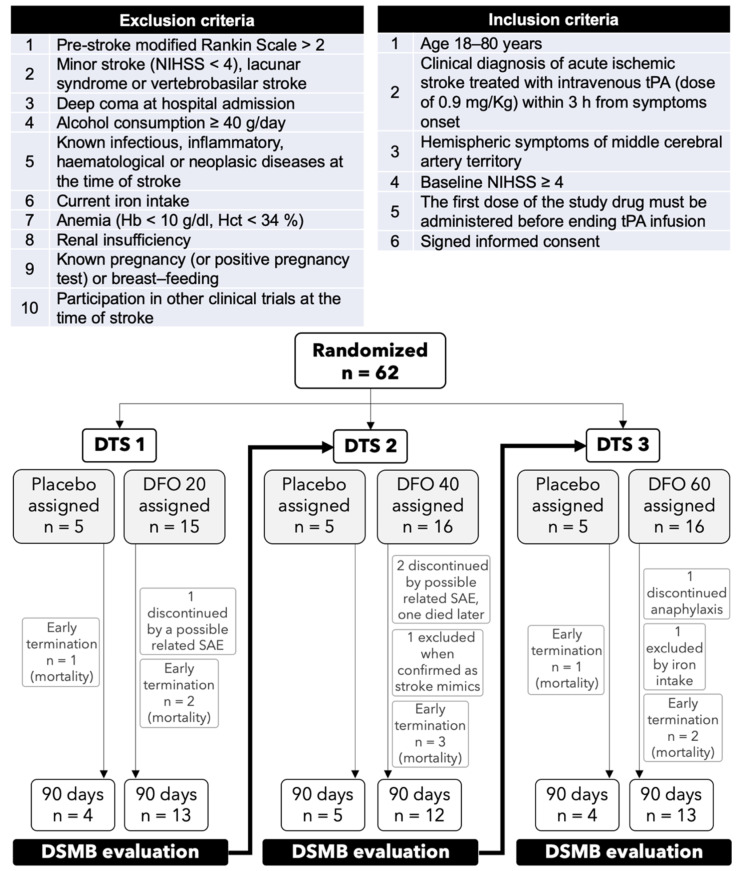
Inclusion and exclusion criteria and CONSORT flow diagram of the three-dose tier sub-studies (DTS). In each DTS early termination cases due to mortality, discontinuation due to serious adverse events (SAE) possibly related to treatment, and patients excluded are indicated.

**Study design and participants:** TANDEM-1 (Thrombolysis And Deferoxamine in Middle Cerebral Artery Occlusion study) was a multicenter, randomized, double-blind, placebo-controlled, dose-finding phase II clinical trial approved by the Spanish Drug Agency (eudraCT 2007-0006731-31) and local Ethics Committee, and registered in clinicalTrials.gov as NCT00777140. Consecutive patients with acute ischemic stroke affecting the middle cerebral artery (MCA) territory, with baseline National Institute of Health Stroke Scale (NIHSS) ≥ 4, treated with IV tPA within 3 h of symptoms onset, and with written informed consent were enrolled at four Spanish centers: Hospital Germans Trias i Pujol, Hospital de La Princesa, Hospital Dr. Josep Trueta, and Hospital Clínico de Santiago de Compostela. CONSORT work flow diagram and inclusion and exclusion criteria are depicted in Figure 1. Hypertension, diabetes or dyslipidemia conditions were diagnosed as explained in the legend in Table 1. Alcohol consumption, current smoking habit, or iron supplementation were assessed at inclusion. Vital signs, laboratory parameters and other parameters of interest for the stroke evolution such as pre-stroke Rankin Scale, NIHSS neurological scale at baseline, previous stroke, inflammatory conditions, or time to recanalization treatment (tPA or endovascular) were recorded.

Study interventions and procedures: Previous reports on pharmacokinetics in healthy humans administered at the maximum recommended DFO dose (10 mg/Kg as a bolus) demonstrate an extremely short half-life of DFO [26]. To quickly reach sustained meaningful concentrations in blood, we administered DFO as a bolus followed by a continuous three-day IV infusion of three DFO doses up to a maximum of 60 mg/Kg/day.

The basis for analyzing the safety of up to 60 mg/Kg/day DFO in the preferred intravenous administration route in terms of pharmacometabolism and pharmacokinetics were as follows. Firstly, to never reach the maximal recommended dose in infusion of 15 mg/Kg/hour (Available online: https://www.medicines.org.uk/emc/product/5/smpc (accessed on 12 June 2021)). The continuous infusion of 60 mg/Kg/day results in the administration of 2.5 mg/Kg of DFO each hour of infusion. As 10 mg/Kg DFO was administered as a bolus immediately preceding the infusion, during the first hour of treatment patients receive 10 mg in the bolus + 2.5 mg in infusion = 12.5 mg/Kg DFO, this being close to, but below, the maximum recommended dose. Secondly, according to the recommendations, DFO dosage should be reduced as soon as possible and should not exceed 80 mg/Kg/day. The dose of 60 mg/Kg/day used added to the 10 mg/Kg DFO bolus results in 70 mg/Kg/day during the first 24 h, close to, but again below, the maximum dose allowed. In addition, the large individual differences in pharmacokinetics and pharmacometabolism of DFO reported in the literature further recommend not to use the higher doses allowed.

The clinical trial was performed as three sequential dose tier sub-studies (DTS), DTS1 to DTS3, from lowest to highest DFO dose. Patients received IV tPA and were randomized using a computer-generated number sheet in a 3:1 ratio to receive either IV DFO or IV placebo treatment starting within the one-hour tPA infusion with no stratification techniques since primary outcome was safety. Some patients received rescue endovascular treatment according to the local protocols. Dose tier protocol was: for DTS1, 0.9% saline solution as placebo (*n* = 5) or a 10 mg/Kg DFO bolus + continuous infusion of 20 mg/Kg/day DFO (*n* = 15); for DTS2, saline as placebo (*n* = 5) or a 10 mg/Kg DFO bolus + continuous infusion of 40 mg/Kg/day DFO (*n* = 16), and for DTS 3, saline as placebo (*n* = 5) or a 10 mg/Kg DFO bolus + continuous infusion of 60 mg/Kg/day DFO (*n* = 16). At the end of the study, 15 placebo patients had been included. The maximum dose per hour never reached the maximal recommended dose in infusion of 15 mg/Kg/hour (Available online: https://www.medicines.org.uk/emc/product/5/smpc (accessed on 12 June 2021)). Trial drug was prepared according to Good Manufacturing and Clinical Practices and masking process and drug randomization was held centralized in the Pharmacy Service of one of the participating sites. Drug trial preparation was done by local non-blinded nurses who signed a confidentiality agreement. Containers and venous and urinary catheters were opaque to prevent viewing of the particular color of the drug and urine in order to maintain the blinding plan.

Safety stopping rules were prespecified as either the presence of 15% of patients (*n* = 3) with symptomatic intracranial hemorrhage (sICH) or 25% mortality (*n* = 5) or the presence of unexpected serious adverse events (SAE) in the DFO arm in one DTS. After each DTS an independent data safety monitoring board (DSMB) reviewed all SAE. The next DTS only started after a positive DSMB evaluation, otherwise termination of the study was mandatory.

Patients were continuously monitored in the stroke units of participating centers. Neurological examination was assessed using the NIHSS by certified neurologists at admission and during hospitalization at 24, 48, and 72 h, and also at 7 and 90 days. Stroke worsening was considered at least a four-point increase in the widely used and reported scale NIHSS score. Follow-up CT scans were performed at 24–36 h after tPA administration to assess intracranial hemorrhage (ICH) and hypodensity volume. Functional outcome was evaluated using the modified Rankin Scale (mRS) at 7 days and 90 days.

Serum was obtained before, and at several time points after DFO administration and was stored at −80 °C. Samples were taken longitudinally from each patient before (baseline), right after the bolus administration, and at different times after the infusion treatment with placebo or DFO, including sampling at 24 and 72 h after the baseline pre-sampling at exactly the same time of day to avoid circadian interferences in the variables under study. At completion of the study, we determined serum levels of DFO (time course along 72 h; *n* = 5–7 patients in each DTS), % of iron saturation of blood transferrin (TSAT) (in patients with serum available pre-, 24 and 72 h post treatment) and ferritin (at admission and at 24 and 72 h) respectively, using an HPLC-based modification of a previously described protocol [27], a method that allows a direct and accurate measure of TSAT in serum samples [12], or an immunodiagnosis ELECSYS 2010 System. To determine TSAT, 0.26 µL of human serum were loaded in Precast 6% TBE urea gels (U-PAGE) (Life Technologies). Human ATf (hATf) and human holotrasferrin (hHTf) from Sigma-Aldrich were used as electrophoretic standards in U-PAGE, ATf, monoferric and diferric hHTf molecules show different electrophoretic mobility in those gels, allowing to detect the amount of each form. Gels were electroblotted onto PVDF-LF membranes (Millipore) which were incubated overnight at 4 °C with the specific anti-transferrin primary antibody and thereafter with the NIR-conjugated secondary antibody. Bands were measured using an Odyssey imaging system and its dedicated software. The anti-transferrin antibody used equally recognizes ATf and iron-containing Tf forms in WB. In contrast with the usual indirect methods, we calculate TSAT (%) directly by combining electrophoretic U-PAGE (that separates Tf into ATf (devoid of iron), two monoferric Tf, and the diferric Tf bands) with immunodetecttion and individual band quantification. We calculated % TSAT in serum samples of stroke patients using our U-PAGE/WB results, according to the following formula: TSAT (%) = (0.5 *mFe·Tf + diFe·Tf)*100/(ATf + mFe·Tf + diFe·Tf.

**Outcomes**: Primary end points were any SAE that occurred within 90 days, the presence of sICH in the 24–36 h CT scan according to the ECASS II criteria [28] (neurological worsening of NIHSS ≥ 4 in any bleeding), early neurological worsening (worsening of NIHSS ≥ 4 within 24 h after stroke onset), and mortality at 90 days.

Secondary end points were good clinical outcomes defined as a dichotomized score (mRS ≤ 2) at 7 and 90 days, the percent reduction of NIHSS at 90 days, pharmacokinetics of IV DFO and the effect of DFO on iron saturation of blood transferrin.

**Statistical analysis**: The stopping safety rules were calculated according to the higher confidence interval of the accepted risk of sICH and mortality in the SITS-MOST study [29] assuming that all drug-related SAE will occur in the DFO arm. All patients enrolled in the trial, including those who had treatment discontinuation at any time point, were included in the safety analysis. Proportions between two groups were compared by using the X^2^ test or Fisher’s exact test, as appropriate. Data are expressed as the mean and SD or the median and quartiles. For vital signs, slope (resulting from a linear regression) and maximum increase were used, whereas for most routine laboratory parameters, the change from baseline was used. Groups were compared using the Student’s *t*-test, the Mann–Whitney U test, or independent or repeated measures ANOVA as appropriate. Statistical analyses were performed using SPSS (version 24) or GraphPad Prism (version 8.3); statistical significance was considered at *p* ≤ 0.05.

## 3. Results

### 3.1. Subject Clinical Characteristics

A total of 62 subjects were enrolled, 45 men and 17 women. The percentage of males is high in all the groups and similar among them: 73%, 60%, 75%, and 81% males were present respectively in the placebo pool and in the treatment groups of DTS1, DTS2, and DTS3. Mean ± SD age of the sample was 66 ± 11 years, median (quartiles) of NIHSS score was 14 [9, 20] and time from stroke onset to IV thrombolysis was 130 [100, 160] minutes and to investigational trial initiation 157 [140, 190] minutes.

All patients were treated with the full dose of IV tPA and received at least one dose of the investigational product according to the estimated weight; 58 (93%) subjects completed the 72 h drug infusion. Forty-seven patients received DFO and 15 placebo. All patients completed the follow up. The treatment was kept blind for all patients during the 90 days of the study, except for one patient displaying an allergic reaction.

Table 1 summarizes the demographic and baseline clinical characteristics in both groups of treatment in each DTS, which were similar across treatment subgroups, except in the DTS3 subgroups that showed higher NIHSS (21.5 versus 12) in the placebo subgroup. By pooling all the placebo groups, the basal NIHSS differences among treatment groups disappear.

### 3.2. Safety Data

Intracranial hemorrhage, neurological worsening and mortality are events that associate to the normal evolution of the stroke pathology. No significant differences were found between placebo and DFO groups in any DTS in the total number of reported adverse events (AE) or SAE in the clinical trial (Table 2), indicating no safety concerns of the treatment with DFO. Each DTS terminated without crossing the safety stopping rules. SAE was reported in 4 patients in the placebo arm pooled from the 3 DTS (26% of placebo pool-treated patients) and in 5 (33.4%), 4 (25%), and 4 (25%) of DFO patients in DTS1, DTS2, and DTS3, respectively. Five events were deemed possibly or definitely drug-related by the local investigator, consisting in a sICH, an asymptomatic bradycardia, a symptomatic hypotension, a patient who suffered an early neurological worsening, and an anaphylaxis considered to be an allergic reaction to DFO during the bolus; in the last four events the drug was discontinued. Only 2 of 62 patients, both treated with the lower dose (IV 20 mg/Kg/day DFO), presented sICH during the study. Nine (14.5%) patients died during the 90-day follow-up post-stroke (causes of mortality are shown in Table S1). No differences were found in the early neurological worsening, early and late mortality (Table 2), or hypodensity volume during the 36 h (Figure S1) post stroke-onset between placebo pool and DFO groups.

### 3.3. Hemodynamic Vital Signs and Routine Clinical Laboratory Results

Continuous monitoring of vital signs and also time-course measures of several laboratory parameters were collected during the 72 h drug infusion. DFO administration at the doses of 20 mg/Kg/day and 40 mg/Kg/day did not modify systolic and diastolic blood pressure (Figure S2), although there was a patient in the 40 mg/Kg/day DFO arm who suffered a symptomatic hypotension requiring medical treatment with complete recovery 10 min later without sequelae. The 60 mg/Kg/day DFO infusion was associated with mild systolic blood pressure-lowering effects that were not clinically significant (Figure S2). A trend to increased heart rate which is not clinically relevant was observed in the 60 mg/Kg/day DFO, with a maximum increase of 13 bpm (95% CI, 4.1 to 22.6). Administration of DFO at any dose did not change body temperature or serum glucose levels (Figure S2). No clinically significant safety concerns or differences across treatment groups were observed for clinical biochemistry or hematological parameters.

### 3.4. Ferritin, DFO Pharmacokinetics, and Effects on TSAT

**Figure 2:**
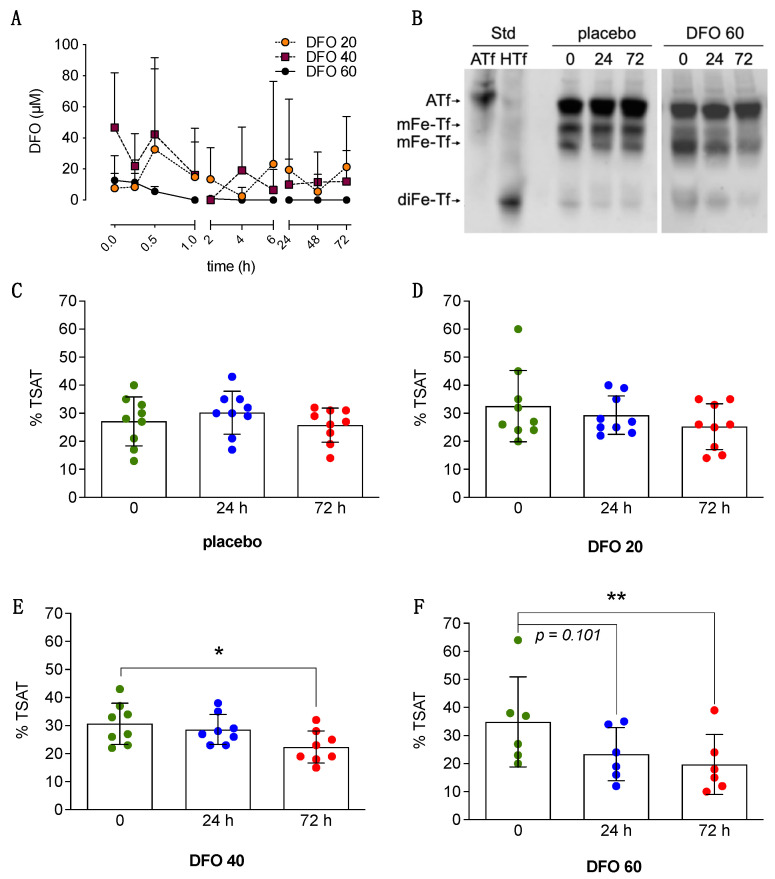
Deferoxamine (DFO) reduces TSAT time- and dose-dependently. (**A**) Time-course of serum DFO levels along the infusion in AIS patients treated with a 10 mg/Kg bolus of DFO IV followed by a 72 h continuous IV infusion of DFO in escalating dose tiers of 20, 40, or 60 mg/Kg/day (blood DFO levels at time = 0 of infusion in this graph are the result of the initial previous 10 mg/Kg bolus of DFO IV). Mean ± SD are shown; no significant effects were found (repeated measures ANOVA). (**B**) U-PAGE/WB depicting the bands of the iron-devoid form of human transferrin standard (Std) (apotransferrin, ATf) and human diferric transferrin (diFe-Tf) standard (holotransferrin, HTf). The iron load of transferrin determines the electrophoretic mobility of the different Tf forms in these urea gels. Arrows indicate the different electrophoretic pattern of ATf, the two monoferric forms of transferrin (mFe-Tf), and the diferric transferrin (diFe-Tf) form in serum samples of stroke patients. Placebo and DFO depict bands of transferrin of two representative patients (one of the placebo group and one of the DFO 60 group) before the onset of treatment (0), and 24 and 72 h after administration. In each lane, optical density of the bands allow calculation of the % TSAT for a given patient at a given time point using the formula: TSAT (%) = (0.5 *mFe·Tf + diFe·Tf)*100/(ATf + mFe·Tf + diFe·Tf). (**C**–**F**) % TSAT before the onset of treatment (0 h), and 24 and 72 h after administration of placebo (**C**), 20 mg/Kg/day DFO (**D**), 40 mg/Kg/day DFO (**E**), or 60 mg/Kg/day DFO (**F**). Values are presented as mean ± SD. * *p* ≤ 0.05, ** *p* ≤ 0.005 (repeated measures one-way ANOVA plus the post-hoc Benjamini–Krieger–Yekutieli test).

Large individual differences in blood DFO levels were observed among patients within a given dose tier group. DFO levels in serum were found higher in most subjects within the first 30 min following the initial 10 mg/Kg DFO bolus administration (average values were 21 µM ± 31). No differences were observed in blood DFO levels when comparing patients in the DFO arms DTS1 (*n* = 7), DTS2 (*n* = 5) and DTS3 (*n* = 5) (Figure 2A).

Baseline serum TSAT in ischemic stroke patients was 31.2 ± 11.0, this being consistent with the TSAT levels and variability reported for healthy non-hemochromatosis individuals [30]. TSAT levels remained steady along the 3 days following ischemia onset in the placebo group as stated using a direct method using longitudinal measures performed in each individual pre- and post- treatment (Figure 2C). Repeated measures analysis show that DFO 20 mg/Kg/day did not change TSAT (Figure 2D). DFO 40 and 60 mg/Kg/day reduced by 30% and 40%, respectively, the TSAT when measured 72 h post-treatment onset (Figure 2E,F). At the 60 mg/Kg/day dose, DFO showed a trend to reduce TSAT (*p* = 0.101) after only 24 h of treatment. Ferritin levels in serum showed a 32% increase 72 h post-stroke onset as compared with baseline (*p* = 0.0046), in agreement with a previous study [18], and DFO treatment did not alter ferritin levels.

### 3.5. Clinical Outcome

**Figure 3:**
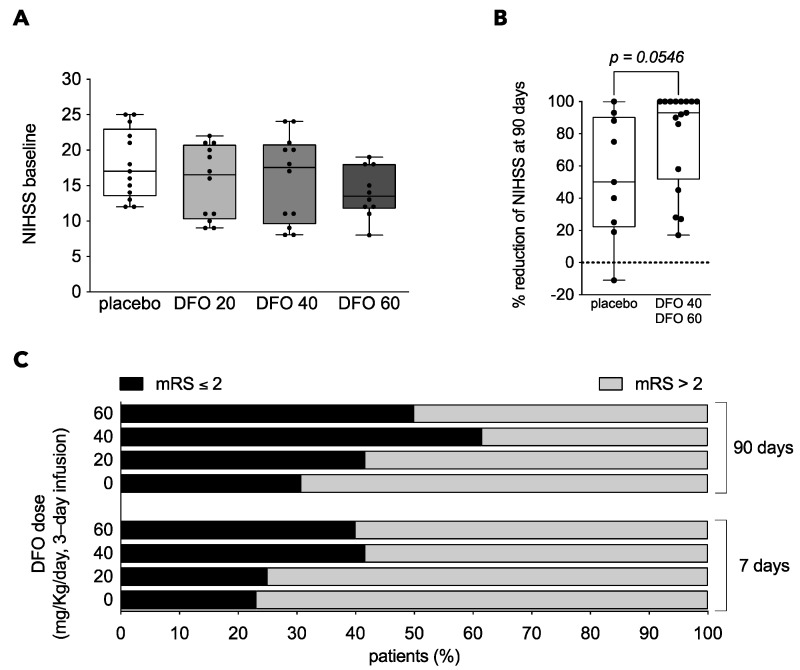
Exploratory analysis to examine whether the TSAT-modifier deferoxamine (DFO) doses favors a good outcome. (**A**) Median and quartiles of baseline NIHSS in a subpopulation of the TANDEM-1 study with NIHSS at admission > 7. Baseline NIHSS were found balanced between the four treatment groups when considering this patient subpopulation (NIHSS > 7) (*p* = 0.24, one-way ANOVA). (**B**) In this subpopulation (NIHSS > 7) we calculated the percentage of neurological improvement with the formula: % reduction of NIHSS at 90 days = ((NIHSS at admission-NIHSS at a given time)*100/NIHSS at admission). As DFO 40 and DFO 60, but not DFO 20, reduce TSAT, these two groups were pooled and compared to the placebo group. We observed that half of the patients in the 40 + 60 DFO group showed a 100% reduction of their initial neurological impairment, in contrast to those in the placebo group (*p* = 0.0546, Mann–Whitney U test). (**C**) Graph depicting the proportion of AIS patients showing good functional outcome (in black) in the placebo and DFO groups. DFO dose tiers of 40 and 60 mg/Kg/day have higher proportion of AIS classified as good outcome patients (mRS ≤ 2) at 7 and 90 days. Values are presented as median and quartiles and compared with one-way ANOVA (**A**) or Mann–Whitney U test (**B**).

No differences were observed in baseline parameters between five of the experimental groups of the study. The lack of patient stratification in the randomization process resulted in a difference in the baseline NIHSS of placebo/DFO arms within the DTS3 (Table 1), this distorting the correct assessment of the effect of treatment within the DTS3 sub-study but not affecting the study when placebo patients were considered as a pool. A post-hoc exploratory analysis was performed to evaluate the trend to outcome improvement of DFO in those patients with moderate-severe ischemic stroke (NIHSS > 7) (*n* = 47) of the whole cohort. Placebo patients in each of the three sub-studies were compiled within a single placebo group, which we term “placebo pool”; NIHSS at admission was not different between placebo and DFO groups in this patient population (Figure 3A). Interestingly, in this patient subpopulation, a trend of reduction of neurological impairment as expressed in percentage of the initial score: (NIHSS baseline-NIHSS 90 days) * 100/NIHSS baseline) was observed in the patient groups administered with the higher DFO doses (DFO 40 and DFO 60, Figure 3B). In addition, a higher proportion of patients having a good outcome (mRS ≤ 2) was observed in the higher DFO dose groups when assessed early (at day 7) or at 90 days after the stroke event (Figure 3C). At day 7, 40% of patients treated with DFO ≥ 40 mg/Kg/day showed a good outcome vs. 23% of patients in the placebo arm. Similarly, at 90 days 50–60% of patients treated with DFO ≥ 40 mg/Kg/day showed a good outcome vs. only 30% in the placebo arm.

## 4. Discussion

This is the first report testing DFO, that targets pro-oxidant iron, in patients with ischemic stroke. DFO as a 10 mg/Kg IV bolus during the tPA infusion followed by a three-day IV continuous infusion of up to 60 mg/Kg/day is safe and well-tolerated, reduces TSAT, and shows a promising trend to better outcome.

We did not find thrombolysis complications due to DFO or any other DFO-induced change in the number and type of adverse events (see Table 2). This safety DFO data in ischemic stroke patients set the groundwork for a larger clinical trial given the recently reported role of iron-induced ferroptosis as a main contributor of brain damage in experimental stroke models [1,4,5,6,31,32,33] and of previous reports in which DFO treatment is associated with lower infarct volume, less mitochondrial free radicals [24], less hemorrhagic transformation, and improved neurological status in experimental ischemic stroke models [4,20,21,24,34,35,36,37]. Of note, either treatment with DFO or prevention of cellular iron uptake have been reported to protect neuronal phenotype cells from excitotoxic cell death through a reduction of oxidative stress [12,38,39].

In iron intoxication/overload, DFO administration for several days as a subcutaneous or intravenous slow infusion is indicated [40]. Although DFO administered systemically does not penetrate very well into the brain through an intact blood–brain barrier (BBB), DFO has been found in the experimentally-induced ischemic brain within the first hour post-stroke [34]. Thus, DFO would preferentially reach the ischemic brain areas, with a leaky BBB, while precluding iron chelation by DFO affecting the activity of cellular metalloenzymes in the healthy brain areas.

Our study provides information about the pharmacokinetics of DFO in ischemic stroke patients, these data being of interest given that in all the species tested so far DFO demonstrate an extremely short half-life in serum/plasma. In healthy humans, IV or i.m. bolus injection of 10 mg/Kg is safe and serum concentrations of DFO range from 5 to 120 µM within minutes after the bolus injection but levels drop quickly [26,41]. To overcome DFO clearance from blood and to secure sustained therapeutic effects during the critical three days post-stroke-onset, the present study used a single 10 mg/Kg bolus of DFO IV, to reach high blood concentrations within minutes, followed by a 72 h continuous IV infusion of 20, 40, or 60 mg/Kg/day DFO. The higher serum DFO levels were observed within the first 30 min of treatment onset as a result of the initial DFO bolus administration (Figure 2A); thereafter, blood DFO levels dropped despite the three-day continuous infusion protocol. We did not find significant differences in DFO pharmacokinetics among different dose tiers along the 72 h follow-up (Figure 2A); this lack of differences could be anticipated in view of: (1) the limited stability and high individual variability of DFO pharmacokinetics reported in a previous clinical study [42], and (2) the rapid clearance of DFO from systemic circulation at clinically relevant doses (half-life of 5–17 min [26,42]). Of note, previous clinical studies using DFO as a continuous infusion in ICH stroke patients did not report pharmacokinetic data [25,43], and a recent study in rats concluded that the biodistribution and clearance from blood was too rapid to generate meaningful blood DFO pharmacokinetics [44].

A dose-dependent effect of the bolus + three-day sustained DFO infusion was observed on the reduction of an important systemic iron parameter, the % saturation of blood transferrin (Tf) with iron (TSAT), which gives the relative amount of iron-free and iron-loaded Tf in blood. Transferrin is the physiological carrier and provider of iron to neurons. We have previously reported the importance of blood Tf load with iron in ischemia events, as iron-loaded transferrin promotes ROS production at reperfusion in stroke models whereas iron-free transferrin prevents the production of 4-hydroxynonenal during ischemia/excitotoxicity in vitro [12]. Blood TSAT remained steady along 72 h in the stroke patients in the placebo group. DFO 40 and 60 mg/Kg/day significantly reduced TSAT by 30% and 40%, respectively, when measured at 72 h (Figure 2E,F) indicating an effective reduction of systemic iron, and the higher 60 mg/Kg/day DFO dose showed a trend to reduce TSAT after only 24 h of treatment (*p* = 0.107, repeated measures analysis). Although DFO is unable to directly remove iron from transferrin at physiological pH (Available online: https://www.medicines.org.uk/emc/product/5/smpc (accessed on 12 June 2021)) [45], TSAT can be modulated by the iron available in blood to bind circulating Tf and/or the iron availability in the Tf-synthesizing cells. In this regard, previous reports have showed that DFO IV infusion rapidly clears around one third of the non-transferrin-bound iron in the blood [46,47] and reduces intracellular labile iron stores in breast cancer cells [48]. The reduction of TSAT by DFO might well be meaningful to the final outcome in the patients according to the fact that: (1) reduction of TSAT by IV administration of iron-free Tf (apotransferrin) at reperfusion has demonstrated neuroprotective and found associated to better outcome in experimental ischemic stroke models [12]; and (2) blood TSAT at reperfusion positively correlated with infarct volume and neurological impairment in experimental stroke; in rats, a 25% reduction in transferrin saturation decreases ischemic brain damage accordingly [12]. In addition, in the placebo group of the present TANDEM-1 study, the endogenous TSAT level seems to impact the outcome of stroke patients, as indicated by preliminary evidence obtained in the present study (Figure S3).

Results in Figure 2F show that a bolus + three-day 60 mg/Kg/day DFO treatment reduces TSAT to half the baseline levels, and the time-effect suggests that administration of 60 mg/Kg/day exceeding three consecutive days would result in too low TSAT (below 16%). Given that TSAT < 16% is usually considered inadequate for erythropoiesis [49], this provides a rationale for the harmful side effects observed associated to the five-day treatment with 62 mg DFO/Kg/day that was initially proposed in the high dose HI-DEF trial protocol [50]. After a three-day high dose DFO treatment, other interventions known to have a mild effect on TSAT, such as a daily intake of the functional compound polyphenol [51], might be worth being evaluated for their possible contribution to TSAT long-term effects and long-term recovery from stroke. Our findings explain both the safety issues of the HI-DEF trial protocol just mentioned and the lack of effect observed when using an alternative intermediate DFO dose (32 mg/Kg/day) and a shorter three-day infusion in the i-DEF [43] and the Chinese Clinical Trial Registry ChiCTR-TRC-14004979 studies [52].

A strength of our study is that we developed and used a direct measurement of TSAT as a reliable surrogate marker of iron status. The effect of each DFO dose was determined longitudinally in each individual patient, from pre-DFO to the end of treatment, each patient being its own control. Using this strategy, we observed that only 40 and 60 mg/Kg DFO reduced TSAT, and we next studied the effect of these TSAT-modifying doses on outcome. The present study focused on safety and it is not powered to statistically assess efficacy or to equally distribute covariates at baseline along experimental groups, this being a limitation of the study. However, an exploratory analysis of efficacy was performed introducing as a selection criteria that of patients with baseline NIHSS > 7, the less severe strokes not being included in this post-hoc analysis and, in addition, we used the percentage reduction of neurological deterioration as an index that considers the specific neurological score of each patient at admission. In this subpopulation in which baseline scores are similar, DFO induced a larger percent reduction of neurological impairment at 90 days (Figure 3B), and the proportion of good outcome patients (mRS ≤ 2) increased with the dose of DFO at 7 and at 90 days. The mRS, that has been used to assess neurological status mostly at 90 days to evaluate long-term outcomes, has been reported in recent papers to be informative in the evaluation of short-term outcomes as well [53,54]. Although this is a post hoc analysis based on a relatively small number of patients, our observations support that DFO, at doses capable of reducing systemic iron and TSAT, seem to be a promising therapy increasing the proportion of patients showing better functional outcome. The effects of DFO preventing neurological impairment induced by AIS does not seem to be due to a possible impact on circulating immune cells since no effect of DFO was observed in blood leucocyte counts in our study (Figure S4), but rather associated with its iron-chelation and antioxidant properties.

## 5. Conclusions

This study demonstrates that DFO in a bolus followed by doses of 40 to 60 mg/Kg/day is safe and well-tolerated in AIS patients, reduces systemic iron over 1–3 days and might provide long term (three months) benefit to AIS patients. Our findings have important implications to select the appropriate DFO dosage for future trials and provide medical plausibility and the rationale to further explore DFO effect in a larger cohort of patients or to test other therapeutic interventions addressed to quickly reduce TSAT in stroke.

## Figures and Tables

**Table 1:** Description of the demographic and baseline clinical characteristics in the placebo and DFO group in each DTS.

KERRYPNX	DTS 1		DTS 2		DTS 3	
	Placebo (*n* = 5)	DFO 20 (*n* = 15)	Placebo (*n* = 5)	DFO 40 (*n* = 16)	Placebo (*n* = 5)	DFO 60 (*n* = 16)
**Age**	64.4 ± 8	67.8 ± 13	67.6 ± 8	64.1 ± 10	60.0 ± 16	70.0 ± 11
**Sex, % male**	40	60	80	75	100	81
**Medical history, % patients**						
Hypertension	80	53	80	50	80	63
Diabetes	60	20	20	19	40	19
Current smoking habit	20	20	20	13	20	6
Dislipemia	40	40	40	31	40	44
Alcohol consumption	20	0	60	19	20	31
Atrial fibrillation	20	40	20	13	20	13
Prior stroke	0	7	0	13	0	19
**Vital signs and laboratory parameters**						
Systolic BP, mmHg	166 ± 41	148 ±21	143 ± 10	140 ± 16	147 ± 16	150 ± 21
Diastolic BP, mmHg	79 ± 9	78 ± 18	80 ± 15	77 ± 11	85 ± 24	80 ± 14
Body temperature, °C	35.8 ± 0.5	36.0 ± 0.3	35.5 ± 0.3	36.0 ± 0.4	35.9 ± 0.6	35.9 ± 0.5
Heart rate, bpm	62 ± 12	82 ± 22	81 ± 19	71 ± 19	94 ± 9.4	70 ± 10
Serum glucose, mg/dL	142 ± 19	107 ± 33	155 ± 71	160 ± 94	212 ± 197	147 ± 72
Platelet count (×1000)	226 ± 61	218 ± 61	277 ± 54	237 ± 75	285 ± 102	234 ± 63
aPTT, s	28 ± 5	26 ± 3	26 ± 6	25 ± 4	28 ± 5	27 ± 3
Hematocrit, %	40.4 ± 3.8	41.4 ± 3.1	45.8 ±3.4	41.0 ± 3.6	45.1 ± 1.1	41.8 ± 4.6
Hemoglobin, g/dL	13.5 ± 1.5	14.0 ± 1.1	15.3 ± 1.0	13.8 ± 1.3	15.3 ± 0.4	14.2 ± 1.5
Creatinin, mg/dL	0.9 ± 0.1	0.9 ± 0.1	0.9 ± 0.5	0.9 ± 0.2	1.1 ± 0.3	0.9 ± 0.2
**NIHSS at baseline**	14	11	16	17	21.5	12
	[12, 15]	[9, 18.5]	[13, 21]	[8, 21]	[17.5, 25]	[7.5, 16.5]
**Stroke subtype, % patients**						
Atherothrombotic	40	7	0	13	40	0
Cardioembolic	20	60	20	44	60	50
Undetermined	40	33	80	31	0	50
Other	0	0	0	6	0	0
**ASPECTS score on baseline CT scan**	10	10	10	10	9.5	10
	[9, 10]	[10, 10]	[10, 10]	[9, 10]	[7.5, 10]	[9, 10]
**Time from onset to tPA, min**	110	136	100	140	132	140
	[90, 110]	[102, 168]	[90, 130]	[95, 155]	[88, 174]	[115, 157]
**Time from onset to trial treatment, min**	140	163	125	170	163	155
	[135, 143]	[150, 213]	[125, 150]	[140, 190]	[128, 192]	[141, 195]
**Rescue endovascular treatment, % patients**	0	7	20	19	80	31

Values are presented as mean ± SD, % percentage, or median [quartiles]. DTS: dose tier sub-study; DFO: deferoxamine (in mg/Kg/day for 3 days); NIHSS: National Institutes of Health Stroke Scale; BP: blood pressure; tPA: tissue plasminogen activator; aPTT: activated partial thromboplastin time. Hypertension is diagnosed if, when it is measured on two different days, the systolic blood pressure on both days is ≥140 mmHg and/or the diastolic blood pressure on both days is ≥90 mmHg. Diabetes, a condition where the body can′t control the amount of glucose in blood, is diagnosed when fasting plasma glycemia is ≥126 mg/dL. Alcohol consumption is here considered as an actual average daily alcohol consumption of less than 40 g/day. Dyslipidemia is diagnosed as an elevation of plasma cholesterol, triglycerides, or both, or a low HDL cholesterol level.

**Table 2:** Safety and outcome results in the placebo and DFO group in each DTS.

	DTS 1			DTS 2			DTS 3		
	Placebo (*n* = 5)	DFO 20 (*n* = 15)	*p*	Placebo (*n* = 5)	DFO 40 (*n* = 16)	*p*	Placebo (*n* = 5)	DFO 60 (*n* = 16)	*p*
Patients with AE, %	100	73.3	0.197	100	75	0.214	100	87.5	0.406
AE	*n* = 13 2.6 ± 1.1	*n* = 28 1.9 ± 1.8	0.349	*n* = 12 2.4 ± 1.1	*n* = 36 2.1 ± 1.8	0.603	*n* = 18 3.5 ± 3.1	*n* = 44 2.7 ± 1.8	0.603
Patients with SAE, %	40	33.4	0.787	0	25	0.214	40	25	0.517
SAE	*n* = 2 0.4 ± 0.5	*n* = 6 0.4 ± 0.6	0.933	*n* = 0	*n* = 6 0.4 ± 0.8	0.445	*n* = 4 0.5 ± 1	*n* = 6 0.4 ± 0.8	0.548
ENW, %	20	20	1	0	6	0.567	0	6	0.567
sICH, %	0	13.3	0.389	0	0	-	0	0	-
Mortality 7 days, %	20	6.7	0.389	0	6.3	0.567	20	12.5	0.676
Mortality 90 days, %	20	13.3	0.718	0	18.8	0.296	20	12.5	0.676

Most stroke patients show adverse events (AE), and a significant number of patients show serious adverse events (SAE) associated to the normal evolution of the stroke pathology. These include symptomatic intracranial hemorrhage (sICH), early neurological worsening (ENW) and mortality. Values are presented as percentage %, *n* and mean ± SD. DTS: dose tier sub-study. DFO: deferoxamine (in mg/Kg/day for three days). The AE and SAE recorded within each DTS allowed to proceed with the next DTS, since each DTS terminated without crossing the safety stopping rules.

## Data Availability

Data is contained within the article and supplementary material.

## Supplementary Materials

The following are available online at https://www.mdpi.com/article/10.3390/antiox10081270/s1, Figure S1: Effect of DFO on CT hypodensity; Figure S2: Effect of deferoxamine (DFO) on cardiovascular parameters, body temperature, and blood glucose; Figure S3: Relationship between % TSAT of each patient in the placebo arm at admission and % reduction of neurological impairment; Figure S4: Effect of 72 h treatment with DFO on blood leucocytes; Table S1: Reported serious adverse events in the placebo and DFO group in each DTS.

## Abbreviations

AE	adverse events
AIS	acute ischemic stroke
aPTT	activated partial thromboplastin time
ASPECTS	Alberta Stroke Program Early CT Score
ATf	apotransferrin
BBB	blood–brain barrier
CT	computed tomography
DFO	deferoxamine
diFe-Tf	diferric transferrin
DSMB	data safety monitoring board
DTS	dose tier sub-study
ENW	early neurological worsening
HTf	holotransferrin
ICH	intracerebral hemorrhage
i.m.	intramuscular
IV	intravenous
MCA	middle cerebral artery
mFe-Tf	monoferric transferrin
mRS	modified Rankin Scale
NIHSS	National Institute of Health Stroke Scale
ROS	reactive oxygen species
SAE	serious adverse events
sICH	symptomatic intracranial hemorrhage; study
TANDEM	Thrombolysis And Deferoxamine in Middle Cerebral Artery Occlusion
Tf	transferrin
tPA	tissue plasminogen activator
TSAT	% of iron saturation of blood transferrin
U-PAGE/WB	urea-polyacrylamide gel electrophoresis/Western Blot
