# Supplementary material for: Targeting Pro-Oxidant Iron with Deferoxamine as a Treatment for Ischemic Stroke: Safety and Optimal Dose Selection in a Randomized Clinical Trial
Source: Antioxidants (Basel). 2021 Aug 10;10(8):1270. doi: 10.3390/antiox10081270 (PMC8389327; doi:10.3390/antiox10081270)
Supplement: Supplementary file 1 [file antioxidants-10-01270-s001.zip › antioxidants-1279447-supplementary.pdf]

Supplementary

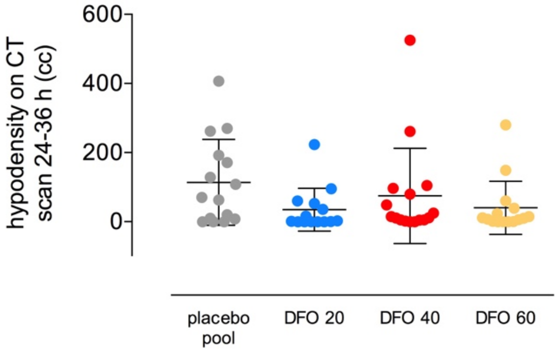

Figure S1. Effect of DFO on CT hypodensity. No effect was observed on hypodensity volume on CT scans during the first 36 h post-stroke onset between placebo and DFO groups.

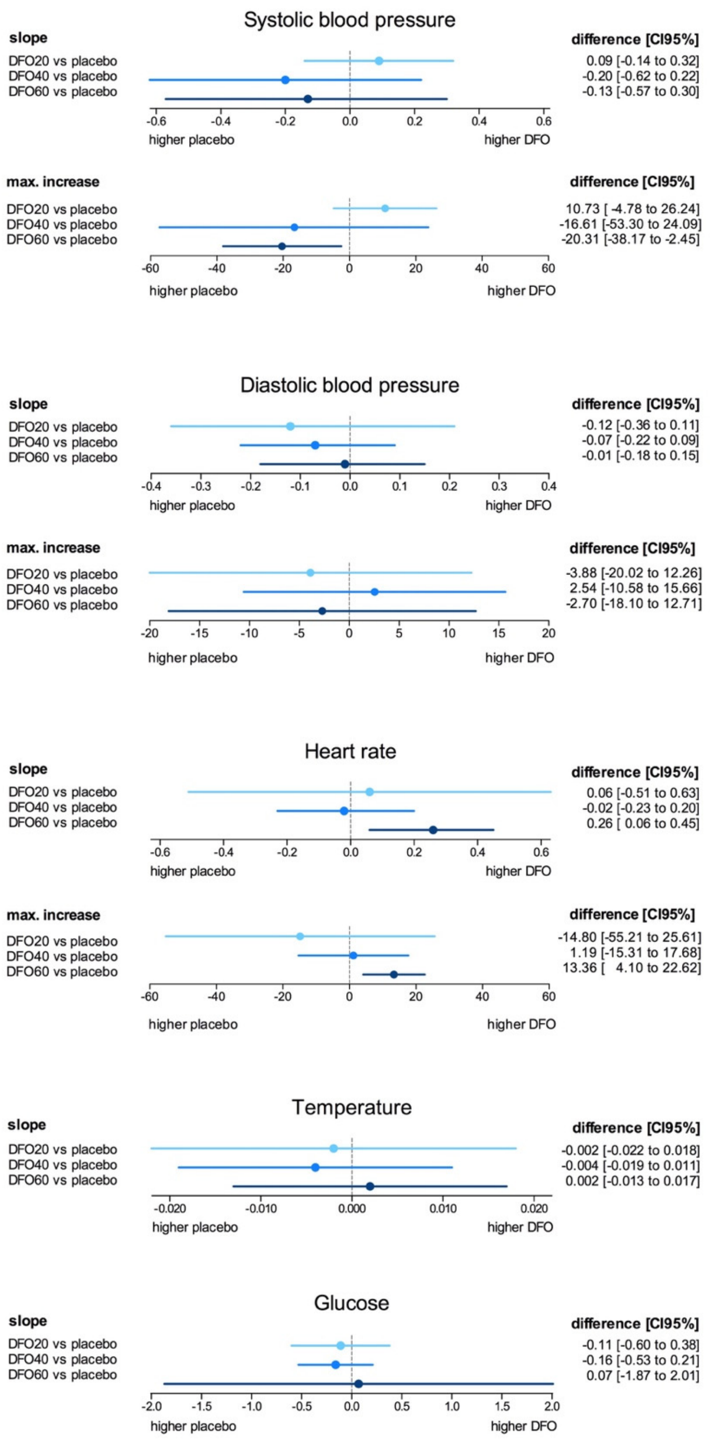

Figure S2. Effect of deferoxamine (DFO) on cardiovascular parameters, body temperature, and blood glucose. Estimated linear regression slope and maximum increase differences for systolic blood pressure, diastolic blood pressure, heart rate, temperature, and blood glucose in each dose tier group of DFO (20, 40 and 60 mg/Kg/day) versus the placebo group (CI 95%). For each patient, a simple linear regression and slope was calculated by plotting in the Y axis values obtained for a given variable at different time points vs time in the X axis. For each patient the maximum increase was calculated as the maximum among the differences between each of the values taken over time and the baseline value.

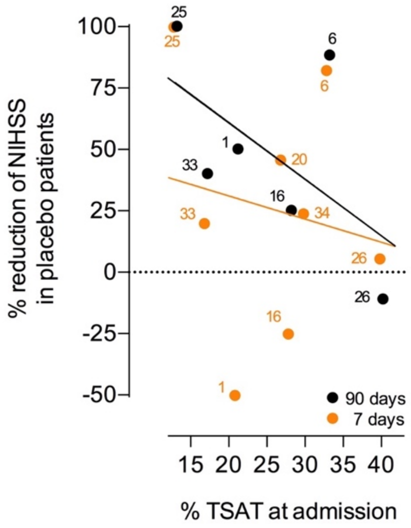

Figure S3. Relationship between % TSAT of each patient in the placebo arm at admission and % reduction of neurological impairment. Serum TSAT was measured at admission and neurological impairment was assessed by an expert neurologist at admission and along the follow-up of the TANDEM-1 clinical trial. Percentage of neurological improvement was calculated at 7 days and at 90 days in the placebo arm using the formula: % reduction of NIHSS in placebo patients=((NIHSS at admission-NIHSS at a given time)*100/NIHSS at admission). Lower endogenous TSAT levels in blood samples obtained in patients at admission associated to a higher improvement of neurological symptoms along time. This trend to improvement in the TANDEM-1 placebo patients is very similar to that we previously observed in experimental animals, in which lower TSAT meant less infarct and less neurological impairment.

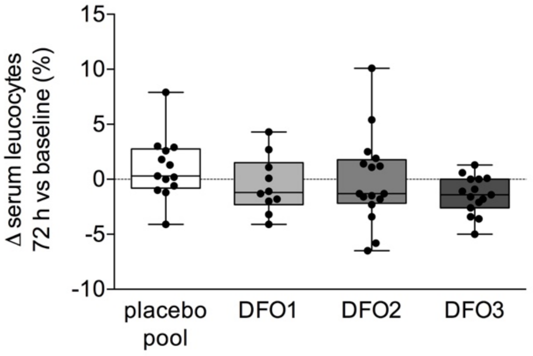

Figure S4. Effect of 72 h treatment with DFO on blood leucocytes. No significant effect of the DFO treatment was observed.

Table S1. Reported serious adverse events in the placebo and DFO group in each DTS

<table>
<tr><th colspan="2">DTS 1</th><th colspan="2">DTS 2</th><th colspan="2">DTS3</th></tr>
<tr><th>Placebo (n=5)</th><th>DFO 20 (n=15)</th><th>Placebo (n=5)</th><th>DFO 40 (n=16)</th><th>Placebo (n=5)</th><th>DFO 60 (n=16)</th></tr>
<tr><td>Neurological worsening
Not related</td><td>sICH- PH2
Not related</td><td></td><td rowspan="3">Deep venous thrombosis
Not related
&
Stroke recurrence
Not related
&
Neurological worsening-
Death
Not related</td><td rowspan="2">Neurological worsening-Malignant MCA infarct
Not related
&
Urinary infection
Not related</td><td>Neurological worsening
Not related</td></tr>
<tr><td>ENW-Malignant MCA infarct
Death
Not related</td><td>sICH-PH2
Possibly related</td><td></td><td>ENW-Malignant MCA infarct
Death
Not related</td></tr>
<tr><td></td><td>Asymptomatic bradycardia
Possibly related-
Discontinued</td><td></td><td rowspan="2">Neurological worsening-Malignant MCA infarct
Death
Not related
&
Broncho-aspiration
Not related</td><td rowspan="3">Broncho-aspiration
Not related
&
Acute Renal Failure
Not related
&
Myocardial infarction-
Death
Not related</td></tr>
<tr><td></td><td>Pulmonary neoplasm-
Death
Not related</td><td></td><td>Symptomatic hypotension
Possibly related-
Discontinued</td></tr>
<tr><td></td><td rowspan="2">Uncontrolled hypertension
Not related
&
ENW-Malignant MCA infarct
Death
Not related</td><td></td><td>ENW-Intracranial hypertension-
Death
Possibly related-
Discontinued</td><td></td></tr>
<tr><td></td><td></td><td>Colon neoplasm-
Death
Not related</td><td></td><td>Anaphylaxis
Definitely related-
Discontinued
#</td></tr>
</table>

DTS: dose tier sub-study. DFO: deferoxamine (in mg/Kg/day for 3 days). Serious adverse effects (SAE) types are reported in the table in bold; those considered to be the cause of death are indicated (text in red) and relationship with treatment (not related, possible-related or related) and drug discontinuation are indicated. Cells in white correspond to patients that suffered only one SAE. Cells displaying identical colour in the table indicate SAE recorded in the same patient: 3 patients presented 2 SAE and 2 patients presented 3 SAE. sICH: symptomatic intracranial hemorrhage. PH2: parenchymal hematoma type. ENW: early neurological worsening. #: Although this patient was randomized into DTS3, anaphylaxis was observed during the 10 mg/Kg DFO bolus administration which is common for the DFO arm in all the DTS.
